# Supplementary material for: Observation of topological transport quantization by dissipation in fast Thouless pumps
Source: Nat Commun. 2020 Jul 27;11:3758. doi: 10.1038/s41467-020-17510-z (PMC7385497; doi:10.1038/s41467-020-17510-z)
Supplement: Supplementary file 1 — Supplementary Information [file 41467_2020_17510_MOESM1_ESM.pdf]

Supplementary Information for

Observation of topological transport quantization by dissipation in fast Thouless pumps

Zlata Fedorova*, Haixin Qiu, Stefan Linden, and Johann Kroha

*Corresponding author: cherpakova@physik.uni-bonn.de

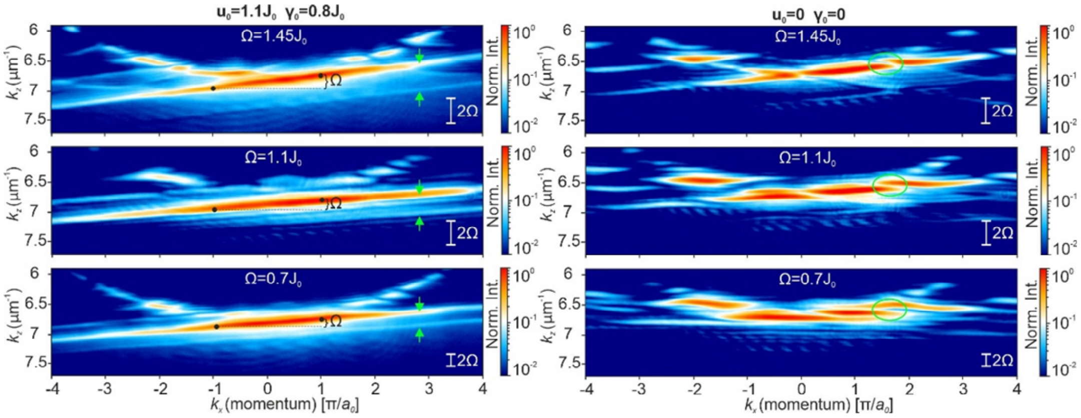

Supplementary Figure 1. Fourier transform in k-space of the data shown in Fig. 6 (a) of the main text. Fourier-space SPP intensity distributions for different driving frequencies and single-site excitation at waveguide A. The images on the left-hand side correspond to the case with cross-section modulation ($u_0 = 1.1J_0,\ \gamma_0 = 0.8J_0$). As the frequency increases the Floquet bands get closer (see arrows) and flatter, nevertheless they remain continuous which is a hallmark of quantized displacement in Real space. On the right-hand side the data for constant cross-section modulation is displayed ($u_0 = 0,\ \gamma_0 = 0$). Here, changing the frequency has an additional effect on the band structure: the gaps at the FBZ boarder start to open (see green circles). Such a behavior of the quasienergies results in the decreasing CoM shift as confirmed by Fig.6 (b). The scale at the bottom right corner in every micrograph shows the size of two FBZs.

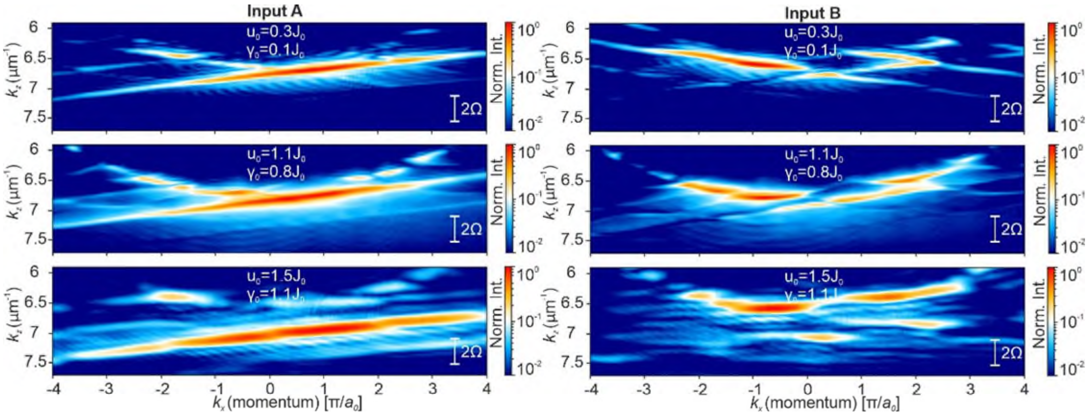

Supplementary Figure 2. Fourier transform in k-space of the data shown in Fig. 7 (a) of the main text. Fourier-space SPP intensity distributions for the arrays with different strengths of the cross-section modulation ($u_0 = 0.3J_0,\ \gamma_0 = 0.1J_0$), ($u_0 = 1.1J_0,\ \gamma_0 = 0.8J_0$), and ($u_0 = 1.5J_0,\ \gamma_0 = 1.1J_0$). Measurements on the left-hand side show the SPP propagation after excitation at sublattice A (low-loss input). Measurements on the right-hand side show the SPP propagation after excitation at sublattice B (high-loss input). The increasing modulation strength has two main effects on the momentum-resolved spectrum: first, the bands get broadened due to higher losses, second, the difference between inputs A and B gets more and more pronounced. In case of the input B the broadening effect is much stronger because of the populated high-loss band.
